# Spatial distribution of rural population using mixed geographically weighted regression: Evidence from Jiangxi Province in China

**DOI:** 10.1371/journal.pone.0250399

**Published:** 2021-04-26

**Authors:** Liguo Zhang, Langping Leng, Yongming Zeng, Xi Lin, Su Chen

**Affiliations:** School of Economics, Jiangxi University of Finance and Economics, Jiangxi, China; Northeastern University (Shenyang China), CHINA

## Abstract

On the basis of the spatial panel data of 2000, 2005, 2010, and 2015, this study uses a mixed geographically weighted regression model to explore the spatial distribution characteristics and influencing factors of the rural (permanent) population in Jiangxi Province, China. Results show that residents in the county area have a significant spatial positive autocorrelation, especially in the lake and mountain areas and the global Moran’ I index is more than 0.05. The influence of social and economic factors presents spatial homogeneity. The effect of urbanization and per capita disposable income is negative, whereas that of agricultural output value and rural electricity consumption is positive. The influence of climate factors presents spatial heterogeneity. The influence coefficient of rainfall in 2015 ranges from [-0.061, 0.133], which has a negative effect on the southwest mountain areas and a positive effect on the northeast lake areas., The influence coefficient of temperature in 2015 ranges from [-0.110, 0.094], which has a positive effect on the southwest mountain areas and a negative effect on the northeast lake areas. The influence coefficients of wind speed and relative humidity range from [-0.090, 0.153] and [-0.069, 0.130] in 2015 respectively, which further reinforce this effect. Therefore, scholars should pay attention to the universal adaptability of economic and social factors. Moreover, they should consider the spatial difference of climatic factors to promote urbanization following the local conditions. Finally, policymakers and concerned non-governmental institutions should fully understand the sensitivity of the rural population in underdeveloped mountain areas to climate factors to promote their rational distribution.

## Introduction

The study of population distribution has always been a research hotspot in the field of population studies. With the acceleration of urbanization, the imbalance of regional economic development, and the worsening climate environment and frequent natural disasters, the study of population distribution has gradually attracted the attention of scholars and governments and achieved fruitful research results [1,2]. Under the background of “emphasizing the city and neglecting the countryside,” scholars focused on the distribution of the total population and the urban population, leaving the distribution of rural residents underexplored [3–6]. As an agricultural country, China’s rural population accounts for a large proportion, in particular, 39.4% of the total population in 2019. The change of rural population has deeply affected the social and economic development, has become the main contradiction and basic national conditions of rural population development, and is an important push to overcome poverty and a critical factor affecting the process and effectiveness of rural revitalization [7,8].

Throughout the existing population distribution research, scholars mainly conducted empirical analysis from two aspects. The first aspect is the traditional linear regression analysis (ordinary least squares model [OLS]), assuming that the influence of factor on the distribution of population in different regions is homogeneous with no spatial differences [9,10]. However, population distribution has spatial characteristics. Its spatial distribution is not random but presents a spatial correlation, and its influencing factors also have spatial characteristics. The second aspect is geographically weighted regression (GWR) analysis, assuming that the degree of influence of each influencing factor varies with the change of spatial position [11–13]. Population distribution has many influencing factors. Some have spatial homogeneity on population distribution and are suitable for traditional linear regression analysis. Others have spatial heterogeneity on population distribution and are fit for GWR analysis.

The research on population distribution has been fruitful, which has laid a solid foundation for this study. However, only a few studies have fully distinguished the spatial homogeneity and spatial heterogeneity of the influencing factors. The mixed GWR model (MGWR) is an organic combination of linear regression and GWR models. It can solve the excessive problems of “spatial homogenization” from the traditional linear regression model and “spatial differentiation” from the GWR model. At present, the study of population distribution by the MGWR model is rare. Thus, the current study intends to use this model to explore the distribution law of the rural population. In addition, the existing population distribution studies usually choose social and economic factors as influencing factors. Only a few studies consider climate considerations but do not take into account spatial heterogeneity [14–17]. Climate conditions provide a geographical framework for population distribution, and its influence on population distribution cannot be ignored.

Jiangxi Province is a traditional agricultural area in China with a large rural population. Relevant data show that rural residents in the province accounted for 42.6% of the total population in 2019, and the climate and topographic conditions in Jiangxi Province are complex and representative in rural population distribution and climate change. Accordingly, this study selects the rural (resident) population in Jiangxi Province as the research object, considers relevant climate factors, and uses the MGWR model. This study also aims to clarify the spatial homogeneity or spatial instability of each influencing factor, analyze the influence of each influencing factor on the distribution of rural residents, and understand the spatial distribution law of the rural population. Our results can provide micro-support for the constant promotion of urbanization, the continuous deepening of poverty alleviation, and the implementation of rural revitalization.

## Research method and data sources

### Research method

#### Spatial autocorrelation analysis

Tobler’s first law emphasizes that spatial correlation is common and evident in nearby places [18]. Scholars commonly used Moran’s I index and Geary’s C index to detect the existence of spatial correlation and spatial agglomeration [19,20]. Accordingly, we use Moran’s I index in spatial autocorrelation analysis in this study. The formulas are as follows:

$$I=\frac{n\sum_{i=1}^{n}\sum_{j\neq i}^{n}w_{ij}(x_{i}-\bar{x})(x_{j}-\bar{x})}{\sum_{i=1}^{n}\sum_{j\neq i}^{n}w_{ij}\sum_{i=1}^{n}{\left(x_{i}-\bar{x}\right)}^{2}},$$ (1)

where *n* represents the total number of studied regional space units, *w_ij_* represents the spatial weight matrix, *x_i_* and *x_j_* respectively represent the attribute value of space unit *i* and space unit *j*, and $\bar{x}$ represents the average value of all attribute values for indicators. The value of Global Moran’s I ranges from −1 to 1 wherein the closer the value (greater than 0) is to 1, the stronger the positive correlation; that is, the cluster of spatial units with the same attributes (high and high adjacent or low and low adjacent). By contrast, the closer the value (less than 0) is to −1, the stronger the negative correlation; that is, the cluster of spatial units with different attributes (the high value is adjacent to the low value). A value close to 0 indicates that the spatial units are irrelevant.

#### MGWR model

A traditional linear regression model assumes that the spatial distribution of sample points has no spatial correlation under the condition that the residual terms are independent; that is, the influence of the respective variables on the dependent variables is uniform. The independent variables are called global variables, and the regression parameters are called constant parameters. When the variables are spatial data, and a spatial correlation exists, the constant parameter coefficients obtained by the traditional linear regression will not reflect the spatial non-stationarity of the variables. The GWR model embeds the 2D coordinates of the sample point data (*u_i_, v_i_*) into the regression parameters to quantify the spatial non-stationarity of the independent variables [21]. In the current study, the independent variables are the local variables, and the regression coefficients are the variable parameters based on spatial positions.

However, in practical problems, the independent variables often contain global and local variables. The influence of some variables is spatially homogeneous, or its spatial non-stationarity is extremely small or even negligible. In this case, the traditional linear regression model is the most suitable one. Other variables have obvious spatial non-stationarity and are fit for GWR modeling. The MGWR model, including the global variables of constant parameter coefficients and the local variables of variable parameter coefficients (varying with geographical location), can solve the excessive problems of “spatial homogenization” from the traditional linear regression model and “spatial differentiation” from the GWR model [22] The formula is as follows:

$$y_{i}=\sum_{j=1}^{h}\alpha_{j}x_{ij}+\beta_{0}(u_{i},v_{i})+\sum_{k=h+1}^{m}\beta_{k}(u_{i},v_{i})x_{ik}+\varepsilon_{i},$$ (2)

Where *y_i_* and *x_ij_* (*i* = 1,2,…,*n*; *j* = 1,2,…,*m*) respectively represent observable dependent variable and independent variables; *m* represents number of independent variables, and *h* global variables and (*m−h*) local variables are present; the sample size is *n*, and *ε_i_* represents random error items; and *α_j_* represents constant regression parameters, and *β*_0_(*u_i_, v_i_*) and *β_k_*(*u_i_, v_i_*) are based on the geographical 2D coordinates (*u_i_, v_i_*) of the regression (variable) parameters.

### Data sources

Given that the data of rural (resident) population in Jiangxi Province only have the statistics of population census or population spot check year, we base our study on the spatial panel data of 2000, 2005, 2010, and 2015. The data in this study mainly include four parts: rural population data, Jiangxi county climate data, social and economic statistical data, and spatial vector data. Among them, we derive the rural population data from the population census or spot check yearbooks of 2000, 2005, 2010, and 2015 (https://data.cnki.net/). The county climate data of Jiangxi Province are obtained by spatial interpolation from meteorological station data of China Meteorological Data Service Center. The data from 834,839,840 and 840 meteorological stations in 2000,2005,2010 and 2015 are obtained, respectively. The meteorological stations monitor the data of temperature, rainfall, wind speed, humidity four times every day, and the daily average data is the average of the timing observations of 2 o’clock, 8 o’clock, 14o’clock and 20 o’clock. Based on this, the annual average meteorological data of meteorological stations are collected in this paper, and the meteorological data of counties and districts are obtained by spatial interpolation and zoning in Arcgis13.0 software. Social and economic statistics from the Jiangxi Statistical Yearbook and the statistical yearbooks of 11 districts in Jiangxi Province (https://data.cnki.net/). And the spatial vector data are obtained from the National Basic Geographic Information Center, mainly include Jiangxi Province county administrative planning boundary, Poyang Lake water body and other shp format maps, which can be loaded on the Arcgis13.0 for editing and processing to analyze the spatial and temporal evolution of rural population.

## Temporal and spatial evolution of rural population distribution in Jiangxi Province

### Regional overview of research

**Fig 1:**
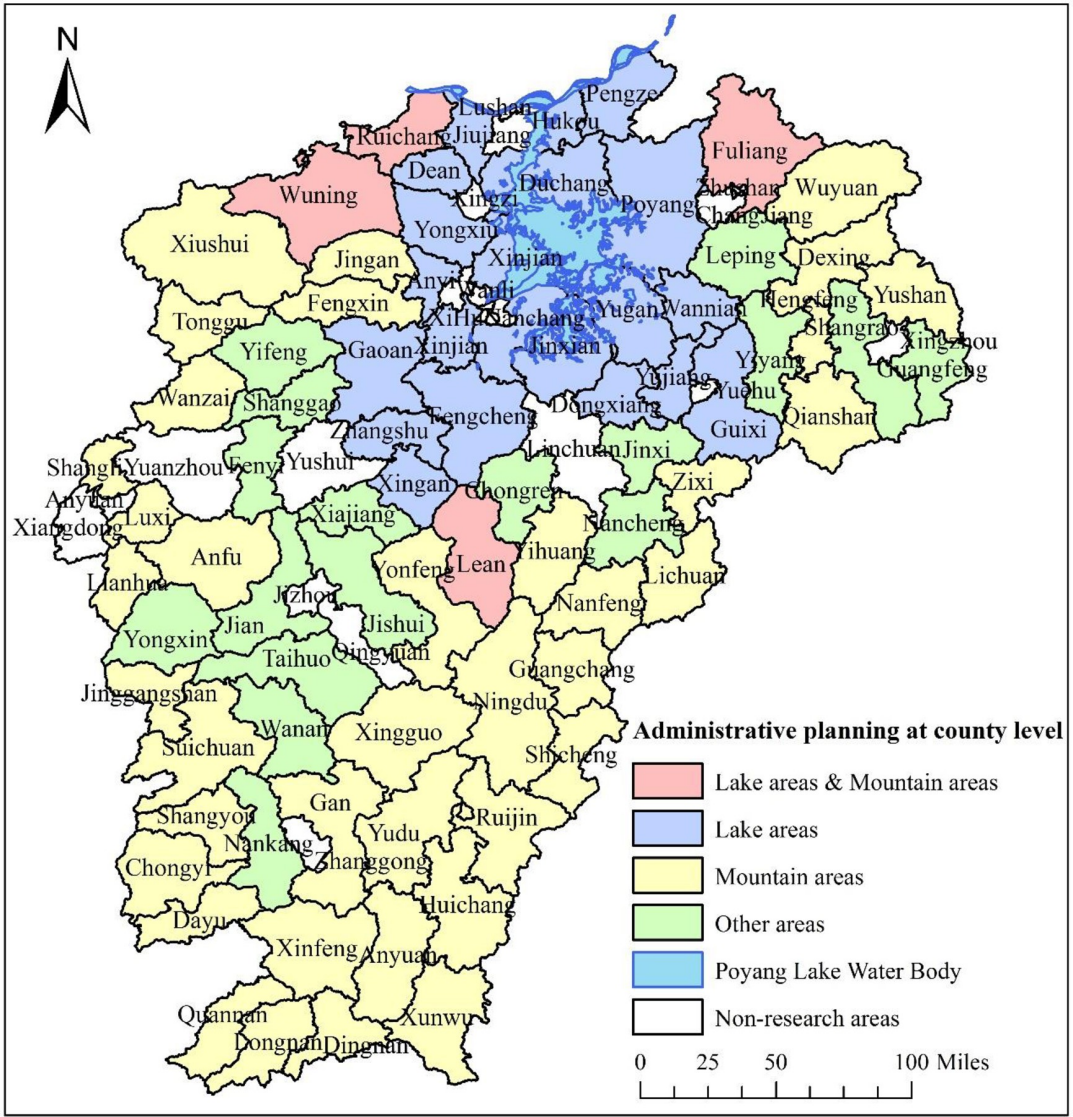
County administrative planning in Jiangxi Province.

Jiangxi Province is located in latitude 24°29’14" to 30°04’41" and longitude 113°34’36" to 118°28’58". The province covers an area of 166,900 km2 and has jurisdiction over 11 prefectural cities and 100 counties (cities and districts). Jiangxi Province is a traditional agricultural area with a considerable rural population. By the end of 2019, a total of 46.661 million permanent residents in 100 counties (cities and districts) are under the jurisdiction of Jiangxi Province, with a rural population of 1,986.8 million accounting for 42.6% of the total population. We consider in this study the high level of social and economic development, the small distribution of rural population, and the lack of some data in 20 municipal districts, such as Qingshanhu District, Qingyunpu District, and Wanli District. Accordingly, we exclude these municipal districts, leaving a total of 80 spatial units for analysis (Fig 1). To explore the spatial differences of the influencing factors on the distribution of rural population in the county area, we divide the spatial research units into three types: lake area, mountain area, and other areas, in accordance with the administrative planning of Poyang Lake Ecological Economic Zone and list of mountain counties (cities) in China County (City) Economic and Social Statistical Yearbook (2012). The lake area includes 25 county spatial units of Poyang Lake water body distribution in Poyang Lake Ecological Economic Zone. The mountain area is in the southern, northwest, and northeast regions of Jiangxi Province, with a total of 42 county spatial units. Wuning County, Ruichang City, Fuliang County, and Le’an County belong to the lake and mountain areas. Different from the lake and mountain regions, the remaining 17 county spatial units belong to other areas. Fig 1 presents the distribution of spatial units in Jiangxi Province.

### Analysis of spatial evolution of rural population distribution in county

**Fig 2:**
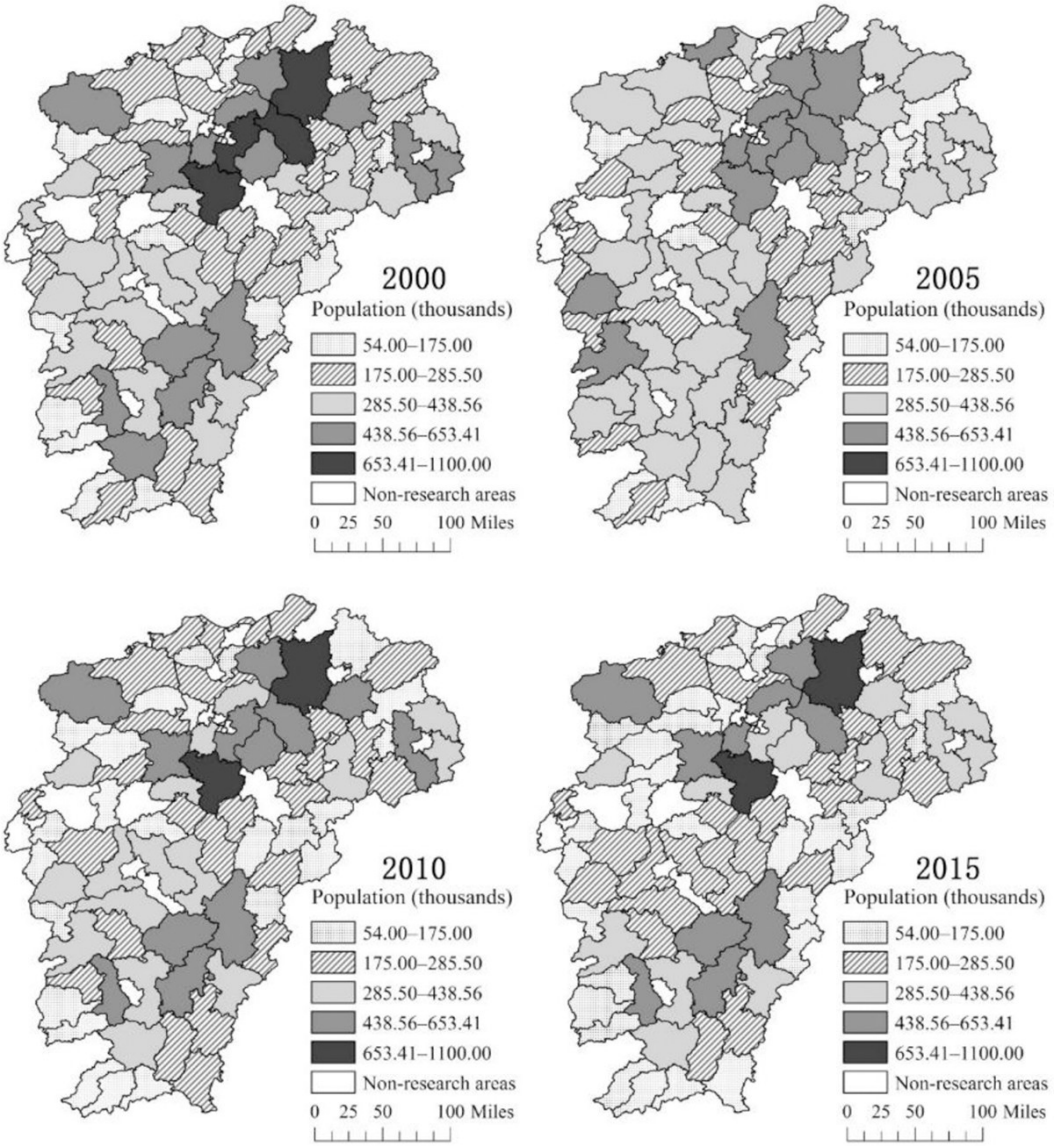
Spatial distribution of the rural population in Jiangxi Province.

**Fig 3:**
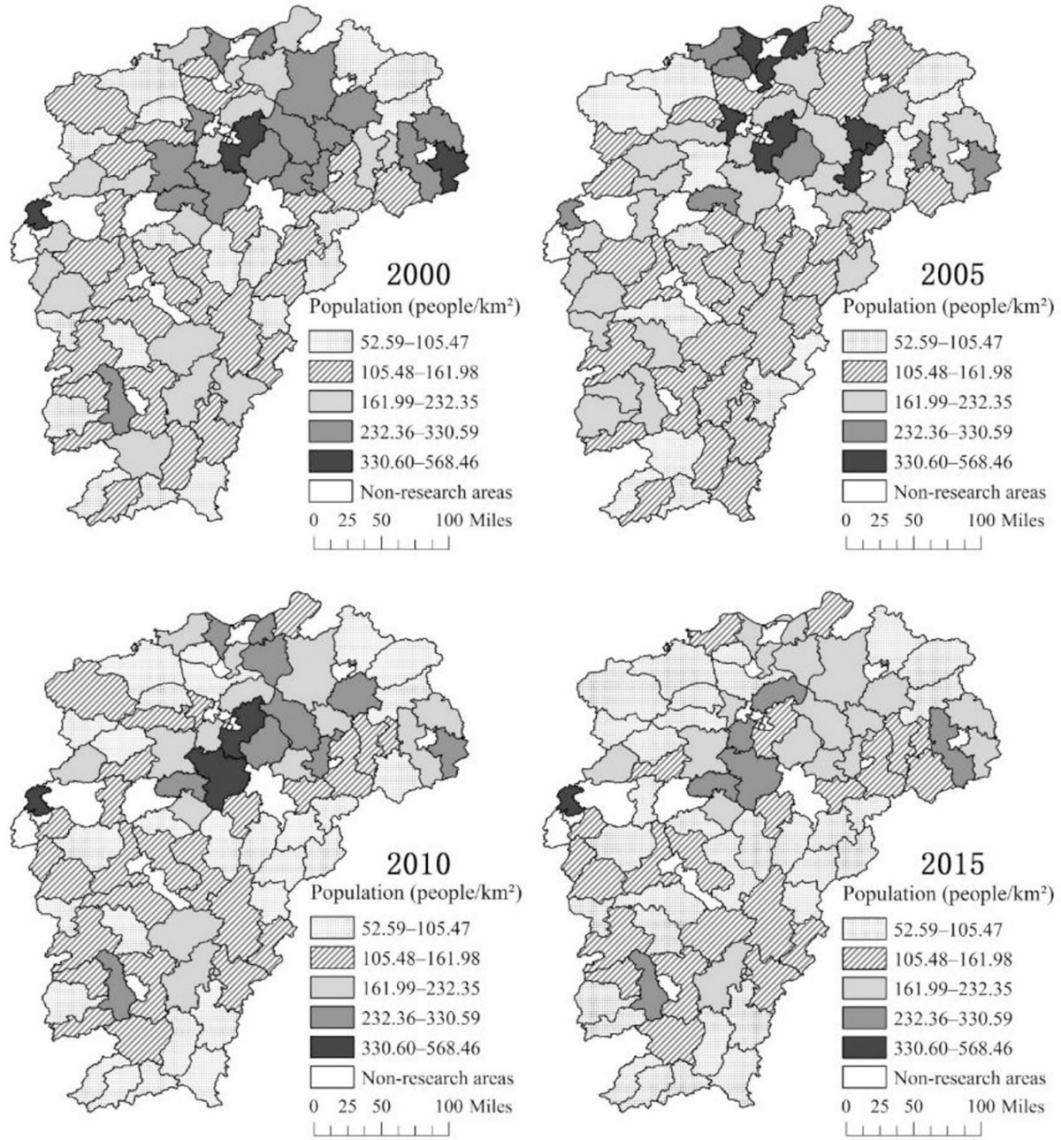
Spatial distribution of the rural population density in Jiangxi Province.

By using ArcGIS software, we draw the spatial and temporal distribution map of the rural population and rural population density in Jiangxi Province in 2000, 2005, 2010, and 2015. We divide the population number and population density into five grades in accordance with the natural breakpoint method. From the distribution of the rural population (Fig 2), since 2000, the number of rural residents in the lake area and the mountain area has been more distributed, and the ecological and economic zone of Poyang Lake and the mountain area of southern Ganzhou have high rural population concentration. In 2000, 2005, 2010, and 2015, the number of counties with a rural population below 285,500 was 46, 32, 51, and 56, respectively. Overall, the rural population in Jiangxi Province shows a trend of increasing first and decreasing later. From the distribution of rural population density (Fig 3), the rural population density in Jiangxi Province shows a decreasing trend in general. In 2000, 2005, 2010, and 2015, the number of counties with a rural population density below 161.99 people/km^2^ was 44, 43, 54, and 56, respectively. Since 2000, the lake area is a concentrated and connected area with high rural population density distribution. However, the rural population density in the lake area has decreased, while the other regions have gradually shown low rural population density agglomeration. In general, the number and density of the rural population in Jiangxi Province show a downward trend, and the lake and mountain areas have a high rural population and high rural population density. Moreover, a certain degree of spatial correlation exists in the distribution of rural residents in Jiangxi Province.

## Spatial correlation and spatial heterogeneity test of rural population distribution

### Spatial correlation test

To verify the spatial correlation of the rural population in Jiangxi Province, we use the global spatial autocorrelation analysis method to calculate the Moran’s I Index of rural population density in 2000, 2005, 2010, and 2015. The density of the rural population in Jiangxi Province in the above years is significantly positive at the significant level of 1%, as shown in Table 1. This finding indicates that a significant spatial positive autocorrelation exists in the distribution of rural residents in Jiangxi Province.

**Table 1:** Global Moran’s I index.

Year	Moran’s I	Z-value	P-value
2000	0.053	4.753	0.000
2005	0.083	6.872	0.000
2010	0.052	4.602	0.000
2015	0.051	4.539	0.000

### Spatial heterogeneity test

We conduct regional mixed OLS regression and panel regression to verify the spatial difference of the influence factors on the distribution of the rural population and divide the influencing factors into global and local variables.

#### Selection of variables

Population density is the most commonly used index for social science research and academic recognition [23]. Therefore, we select rural population density as the dependent variable in this study to measure the distribution of the rural population in Jiangxi Province. In the selection of independent variables, many factors affect population distribution. In this study, we divide the influencing factors into climatic and social and economic factors. Following previous studies, we consider annual average rainfall (Rain), annual average temperature (Tem), annual average wind speed (Wind), and annual relative humidity (Hum) the four most common climatic indicators for climatic factors [24,25]. On the basis of existing studies and the actual situation and data availability in Jiangxi Province, we select urbanization level (Urban), per capita disposable income of rural residents (Income), the output value of agriculture, forestry, animal husbandry, and fishery per unit area (PaO), and rural electricity consumption per unit area (PaE) as variables for social and economic factors [26–28]. Table 2 shows the specific indicators selected in this study.

**Table 2:** Selection of variables.

Variables		Index	Abbreviation	Unit
**Dependent Variable**		Rural Population Density	RPD	people/km2
**Independent Variables**	**Climatic Factors**	Annual average rainfall	Rain	mm
		Annual average temperature	Tem	°C
		Annual average wind speed	Wind	10−1 m/s
		Annual relative humidity	Hum	%
	**Social and Economic Factors**	Level of urbanization	Urban	%
		Per capita disposable income of rural residents	Income	RMB/person
		Output value of agriculture, forestry, animal husbandry, and fishery per unit area	PaO	104 RMB/km2
		Rural electricity consumption per unit area	PaE	kWh/km2

#### Mixed OLS regression and panel regression

We treat all variables logarithmically to eliminate heteroscedasticity. Accordingly, we determine the influence of each variable on elasticity. Table 3 shows the regression results. The goodness of fit of each model has reached a high level, and their fitting effect is good. By comparing the variable coefficients and significance levels of global and sub-regional regression, we can obtain the following findings. (1) The influence of climatic factors on the distribution of the rural population in different regions varies greatly. The influence of climatic factors on the distribution of the rural population in the mountain area is not significant. The distribution of the rural population in the lake area and other regions is sensitive to climate change. The influence degree of climatic factors on the distribution of rural populations in other regions is different in direction. (2) Social and economic factors have little influence on the distribution of the rural population in different regions. The effect of urbanization level (Urban) and per capita disposable income of rural residents (Income) on the distribution of the rural population in different regions is significantly negative. The output value of agriculture, forestry, animal husbandry, and fishery per unit area (PaO) and rural electricity consumption per unit area (PaE) are significantly positive for the distribution of the rural population in different regions. The influence coefficient of each economic and social factor is not different. The Lagrange multiplier test shows that the results of Moran’s I, LMlag, and LMerror and its robustness tests R-LMlagand R-LMerror pass the significance test at a significant level of 1%. This finding strongly rejects the original hypothesis of “no spatial autocorrelation” and further verifies the necessity of spatial measurement analysis.

**Table 3:** Mixed OLS regression and panel regression results.

	Global Regression		Lake Area		Mountain Area		Other Areas	
	OLS	Panel	OLS	Panel	OLS	Panel	OLS	Panel
**Rain**	−0.042 (−0.28)	−0.178 (−1.20)	−0.452 (−1.45)	−0.524 (−1.60)	−0.092 (−0.45)	−0.238 (−1.17)	0.199 (0.68)	0.036 (0.13)
**Tem**	−0.395* (−1.69)	−0.342 (−1.17)	−0.847** (−2.32)	−0.655 (−1.56)	−0.349 (−0.90)	−0.057 (−0.11)	3.256*** (3.65)	1.966* (1.96)
**Wind**	0.358** (2.02)	0.354* (1.93)	−1.069*** (−2.91)	−0.863** (−2.29)	0.415* (1.74)	0.381 (1.53)	1.201*** (2.86)	0.706* (1.69)
**Hum**	−0.413 (−0.63)	−0.884 (−1.52)	−3.754*** (−2.72)	−3.877*** (−2.97)	−0.155 (−0.19)	−0.704 (−0.95)	2.968* (1.72)	1.449 (1.02)
**Urban**	−0.426*** (−8.71)	−0.422*** (−9.25)	−0.545*** (−5.73)	−0.540*** (−5.96)	−0.417*** (−6.88)	−0.393*** (−6.63)	−0.478*** (−4.04)	−0.423*** (−4.51)
**Income**	−0.468*** (−9.54)	−0.382*** (−7.41)	−0.344*** (−4.17)	−0.288*** (−3.35)	−0.456*** (−7.30)	−0.362*** (−5.18)	−0.678*** (−4.82)	−0.452*** (−3.20)
**PaO**	0.560*** (13.52)	0.491*** (10.17)	0.563*** (7.58)	0.495*** (6.18)	0.541*** (8.77)	0.444*** (6.11)	0.562*** (4.45)	0.449*** (3.30)
**PaE**	0.167*** (5.64)	0.125*** (4.02)	0.129** (2.16)	0.134** (2.12)	0.176*** (4.52)	0.130*** (3.07)	0.266*** (4.59)	0.118*** (1.96)
**C**	8.514** (2.48)	11.761*** (3.50)	32.328*** (4.38)	32.208*** (4.38)	7.410** (1.70)	10.473** (2.37)	−20.644** (−2.14)	−7.335 (−0.87)
**R2**	0.681	0.789	0.718	0.85	0.642	0.766	0.579	0.624
**N**	320	320	100	100	168	168	68	68
			**Statistics**	**P-value**			**Statistics**	**P-value**	
**Global OLS LM Test**	**Moran’s I**		19.019***	0.000				
	**LMerror**		35.432***	0.000		**LMlag**	9.828***	0.002
	**R-LMerror**		32.551***	0.000		**R-LMlag**	6.947***	0.008

**Note:** All variables are logarithmic. The symbols *, **, and *** indicate significance at levels of 10%, 5%, and 1%, respectively.

The spatial correlation test and spatial heterogeneity test results show that a significant spatial correlation exists in the distribution of the rural population in Jiangxi Province. The effects of social and economic factors and climatic factors on the distribution of the rural population in Jiangxi Province show spatial homogeneity and spatial heterogeneity, respectively. The traditional linear regression and GWR are not applicable. Therefore, we use social and economic factors as global variables and climatic factors as local variables in this study to conduct a spatial measurement analysis of MGWR.

## Analysis of the driving factors of rural population distribution in Jiangxi Province

### MGWR results

We compare the results of the global OLS model and the MGWR model in this study (Table 4). The goodness-of-fit, AIC (Akaike information criterion) value (the smaller, the better), and residual squared sum (the smaller, the better) of the MGWR model in 2000, 2005, 2010, and 2015 are better than those of the global OLS model to some extent, indicating that the former is superior to the latter.

**Table 4:** Comparison of the OLS regression model and the MGWR model results.

Year	Model	R^2^	AIC value	Residual Squared Sum
**2000**	**OLS**	0.729	79.321	4.463
	**MGWR**	0.742	17.662	3.988
	**Improvement**	0.013	61.659	0.475
**2005**	**OLS**	0.589	38.975	5.706
	**MGWR**	0.628	34.426	4.755
	**Improvement**	0.039	4.549	0.951
**2010**	**OLS**	0.794	3.850	3.670
	**MGWR**	0.835	−9.556	2.651
	**Improvement**	0.041	13.406	1.019
**2015**	**OLS**	0.759	9.565	3.951
	**MGWR**	0.789	6.289	2.986
	**Improvement**	0.030	3.276	0.965

The goodness-of-fit of the MGWR model in 2000, 2005, 2010, and 2015 has reached a high level of 0.742, 0.628, 0.835, and 0.789, respectively (Table 5) and the adaptive bandwidth, which is 1.85,1.49,1.43 and 1.18 in 2000,2005,2010 and 2015, respectively. We divided the above results into two parts: local and global variables. Regarding local variables, the regression coefficient of variables follows the geographical location of the spatial units. The spatial influence coefficient of each local variable is quite different, and the degree of influence of some variables has directional change. Over time, the degree of influence of local climate variables on the spatial units is varied, which further verifies the spatial non-stationarity of the effect of climate factors on the distribution of the rural population. Regarding global variables, the regression coefficients of variables are constant parameters based on spatial geographical location, and the influence of the regression coefficient of each variable on the distribution of the rural population has spatial stationarity. Except for the temperature in 2000, which had no significant effect, the global variables in other years significantly affected the distribution of the rural population. In addition, the influence of variables has no directional change in different years, which further verifies the spatial stability of social and economic factors on the distribution of the rural population. The effect of urbanization level (Urban) on the distribution of the rural population is significantly negative. This finding indicates that an increase in urbanization level will reduce the regional rural population density. With the increase in urbanization level, rural residents will continue to migrate to the cities, and their number will gradually decrease, which is in line with our expectations. The disposable income of rural residents (Income) has a negative effect on the distribution of rural population, and the increase of per capita disposable income of rural residents will reduce the density of the regional rural population. Given that the resource elements in the countryside are limited, the improvement of the per capita disposable income of rural residents mainly comes from the migrant workers of the rural population. With a large number of migrant workers in rural areas, the density of the rural population decreases gradually. The output value of agriculture, forestry, animal husbandry, and fishery per unit area (PaO) has a significant positive effect on the distribution of the rural population. This finding implies that the higher the output value of agriculture, forestry, animal husbandry, and fishery per unit area, the more the distribution of the rural population.

**Table 5:** Estimation results of MGWR model.

Year	Local Variables (Climatic Factors)					Global Variables (Social and Economic Factors)	
	Variables	Mean	Minimum	Median	Maximum	Variables	Coefficient
**2000**	**Rain**	0.069	0.045	0.073	0.083	**Urban**	−0.155*** (−4.602)
	**Tem**	0.007	−0.040	−0.004	0.108	**Income**	−0.057 (−1.573)
	**Wind**	0.058	0.003	0.066	0.086	**PaO**	0.303*** (7.934)
	**Hum**	−0.034	−0.114	−0.039	0.055	**PaE**	0.103*** (2.663)
	**Intercept**	4.988	4.961	5.065	5.081	**R**^**2**^ **Adj.**	0.742
**2005**	**Rain**	0.052	−0.038	0.058	0.091	**Urban**	−0.151*** (−4.799)
	**Tem**	−0.163	−0.218	−0.172	−0.101	**Income**	−0.062* (−1.694)
	**Wind**	−0.052	−0.165	−0.047	0.028	**PaO**	0.225*** (4.908)
	**Hum**	−0.061	−0.181	−0.037	−0.016	**PaE**	0.091** (2.072)
	**Intercept**	5.028	5.019	5.071	5.218	**R**^**2**^ **Adj.**	0.628
**2010**	**Rain**	0.013	−0.132	0.017	0.135	**Urban**	−0.184*** (−7.195)
	**Tem**	−0.015	−0.077	−0.007	0.038	**Income**	−0.075** (−2.506)
	**Wind**	0.058	−0.041	0.069	0.132	**PaO**	0.335*** (9.496)
	**Hum**	0.063	0.022	0.067	0.113	**PaE**	0.126*** (3.730)
	**Intercept**	4.790	4.801	4.847	4.898	**R**^**2**^ **Adj.**	0.835
**2015**	**Rain**	0.025	−0.061	0.028	0.133	**Urban**	−0.089*** (−3.062)
	**Tem**	0.029	−0.110	0.054	0.094	**Income**	−0.135*** (−3.783)
	**Wind**	0.011	−0.090	−0.007	0.153	**PaO**	0.335*** (8.083)
	**Hum**	0.013	−0.069	0.006	0.130	**PaE**	0.145*** (3.372)
	**Intercept**	4.694	4.659	4.765	4.795	**R**^**2**^ **Adj.**	0.789

**Note:** All variables are logarithmic. The symbols *, **, and *** indicate significance at levels of 10%, 5%, and 1%, respectively.

In the case of a certain level of investment and agricultural mechanization, the higher the level of human capital investment, the higher the output value of agriculture, forestry, animal husbandry, and fishery per unit area. The rural population is the main source of agricultural and human capital investment with high regional rural population density. The influence of rural electricity consumption per unit area on the distribution of the rural population is significantly positive; that is, the higher the level of rural electricity consumption in the region, the more the distribution of the rural population. This finding is consistent with our expectations.

### Spatial heterogeneity analysis of climate factors

**Fig 4:**
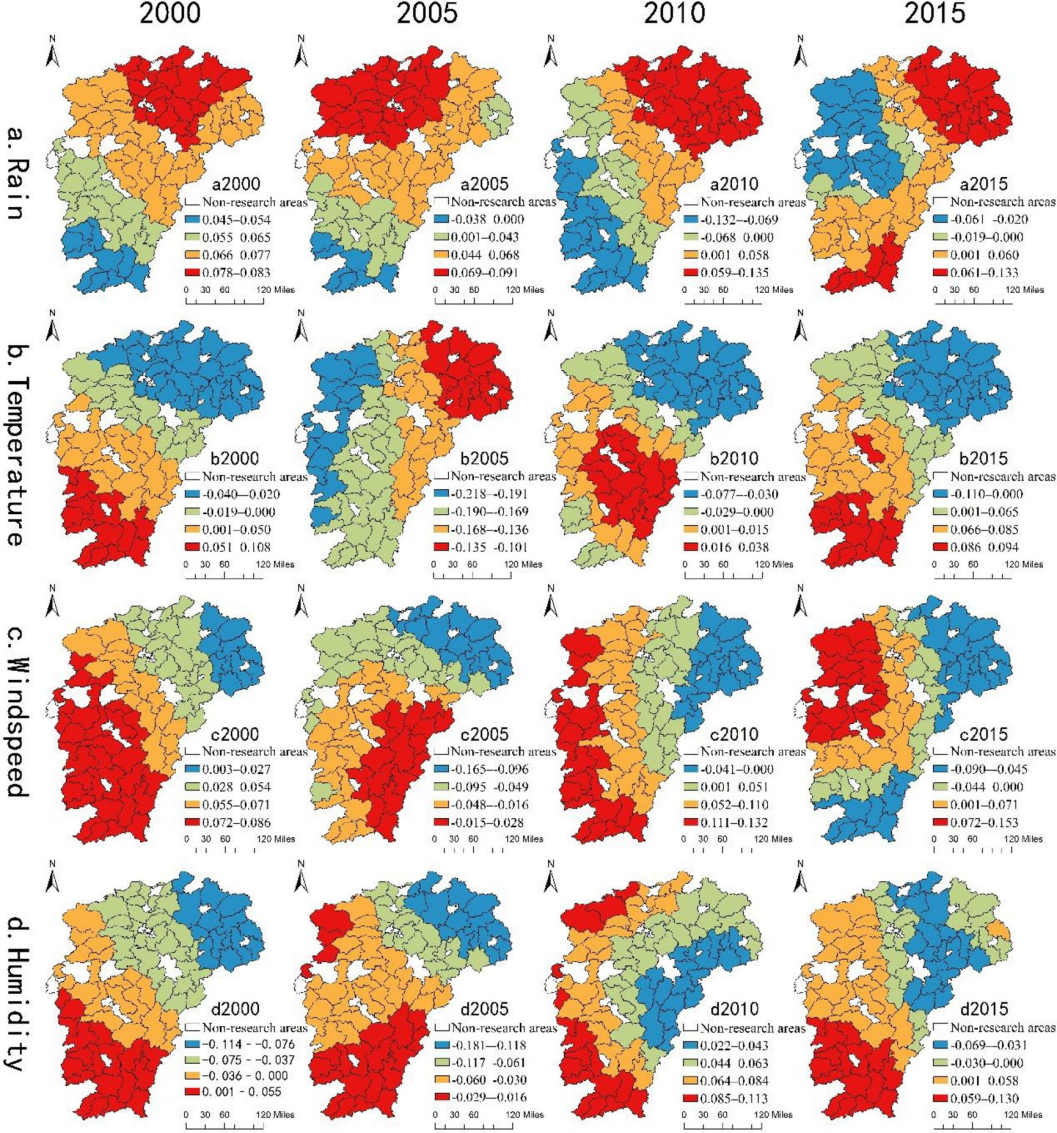
Spatial distribution of regression coefficients of local (climatic) variables.

Climatic factors have spatial heterogeneity in the distribution of the rural population in different regions. To reflect this spatial difference clearly, we use ArcGIS in this study to make a spatial quartile distribution map of four local (climate) variables (Fig 4). Rainfall shows positive and negative effects on the northeast lake and southwest mountain areas, respectively. By contrast, temperature shows negative and positive effects on the northeast lake and southwest mountain areas, respectively. Similarly, wind speed and humidity show negative and positive effects on the northeast lake and southwest mountain areas, respectively, which strengthen the influence of temperature on the regional population distribution to some extent.

(1) Fig 4(A) shows the effect of annual average rainfall on the distribution of the rural population in the region. The annual average rainfall in most areas has a positive effect on the distribution of the rural population, but over time, its negative influence has gradually appeared in some regions. In 2000, the annual average rainfall regression coefficient was greater than 0, and its positive effect on the rural population distribution increased gradually along the southwest of Jiangxi Province to the northeast. The positive effect of rainfall on the rural population distribution in the lake area is greater than that in the mountain area because the county economic development level of Jiangxi Province was not high in 2000, and the rural population income mainly comes from agricultural production. The increase of rainfall could significantly promote agricultural production in the southern region, and the effect of rainfall on the plain area (lake area) is generally higher than that in the mountain area.

(2) Fig 4(B) shows the effect of average annual temperature on regional rural population distribution. The temperature has a negative effect on rural population distribution in northeast Jiangxi Province (mainly lake area) and a positive effect on that in southwest Jiangxi Province (mainly mountain area). The mountain area has low latitude and high altitude. Its annual and daily temperature difference is small, in particular, summer is not too hot, and winter is not too cold in this area. The temperature in mountain area is lower in summer and higher in winter than lake area, which is more suitable for rural population to carry on ecological livable construction. In the case of global warming, not only social and economic factors affect rural population migration flows, but also climatic factors, which make the population move to areas with suitable temperature. Thus, temperature has negative and positive effects in the northeast lake and southwest mountain areas.

(3) Fig 4(C) and 4(D) show the effects of annual average wind speed and relative humidity on the regional rural population distribution, respectively. The negative effect of wind speed on the mountain area in southern Ganzhou and the northeast area of Jiangxi Province has gradually appeared. By contrast, it has a positive effect on the northwest area of Jiangxi Province. The wind speed and relative humidity level strengthen the influence of temperature on the distribution of the rural population in the county area. The temperature in the northeast lake area is lower than that in the southwest mountain area in winter, and the wind speed and humidity level in the northeast lake area are higher than that in the southwest mountain area. The higher the wind speed and relative humidity level, the higher the possibility that the regional somatosensory temperature will be significantly lower than the regional temperature. This condition leads to the cold winter in the northeast lake area, which is not conducive to the construction of ecological livability for the rural population. In the southwest mountain area, the winter temperature is relatively high and coupled with high mountain ranges, which prevent the cold air from driving south. The wind speed and humidity level are relatively low, making the winter weather bearable with less rain and snow, which is suitable for communities. At the same time, the sensitivity of the rural people to climate factors is strong, and moving to areas with suitable climate and a good economic development foundation is easier for them.

The influence of climatic factors on the distribution of the rural population in 2005 was quite different from the overall influence trend reflected in the negative effects of temperature, wind speed, and relative humidity on the distribution of the rural population in the whole province. The reason is 2005 was a year with more meteorological and heavy disasters in Jiangxi Province. The summer temperature was higher than in previous years, the winter weather and snow-related disasters reached a historical record, and the storm disaster points were wide. Moreover, four typhoons: “Begonia,” “Coral,” “Taili,” and “Longwang” entered Jiangxi Province in 2005.

## Conclusion and discussion

By analyzing the data of 80 counties (cities and districts) of Jiangxi Province in 2000, 2005, 2010, and 2015, we provide evidence of the spatial homogeneity or spatial heterogeneity of each influencing factor. We use spatial autocorrelation analysis and the MGWR model to analyze the spatial distribution characteristics and influencing factors of the rural population in Jiangxi Province. We list our main conclusions as follows: First, the distribution of the rural population in Jiangxi Province is concentrated in the lake and mountain areas, showing significant spatial autocorrelation. Second, no significant spatial difference exists in the influence of social and economic factors on rural population distribution. Third, significant spatial differences exist in the influence of climatic factors on the distribution of the rural population. The sensitivity of the rural population to climatic factors in different regions is varied, mainly from the aspects of agricultural production and ecological livability. This study also contributes noble findings to the literature as follows: First, population distribution has spatial attributes, and thus lack of spatial perspective or excessive “spatial differentiation” cannot accurately reflect the characteristics of rural population distribution. Future population distribution research should fully clarify the spatial homogeneity and spatial heterogeneity of various influencing factors. Second, in promoting urbanization and managing rural population distribution, scholars should fully understand the universal applicability of social and economic factors and the spatial differences of climate factors In the process of urbanization, we should realize the role of economic and social development in promoting population urbanization, at the same time, we should fully consider the influence of climate and topographic conditions on the difference of population distribution in different regions, and formulate relevant measures in accordance with the local conditions. Third, when concentrating on poverty alleviation and development in the mountain area with special difficulties, policymakers and concerned non-governmental organizations should pay attention to the high sensitivity of underdeveloped communities to climatic factors. They should also rationally promote the migration and flow of the marginalized population, and, if necessary, transfer them to areas with suitable climate and small geographical costs. The above proposals are critical for the decisive battle against poverty. The deficiency of this study is that the scale of the study is too macro, and the influence of social, economic and climate factors on population distribution can only be analyzed at the county level. The follow-up study will be based on a micro perspective (village or individual) to study the impact of social, economic and climate factors on rural population distribution or population mobility.

## Supporting information

S1 Data (ZIP)

## References

[1] Samson J., Berteaux D., McGill B. J., & Humphries M. M. (2011). Geographic disparities and moral hazards in the predicted impacts of climate change on human populations. *Global Ecology and Biogeography*, 20(4), 532–544. 10.1111/j.1466-8238.2010.00632.x

[2] Barbier E. B., & Hochard J. P. (2017). Poverty, rural population distribution and climate change. *Environment and Development Economics*, 23(03), 234–256. 10.1017/s1355770x17000353

[3] Yue T. X., Wang Y. A., Liu J. Y., Chen S. P., Qiu D. S., Deng X. Z., … et al. (2005). Surface modelling of human population distribution in China. *Ecological Modelling*, 181(4), 461–478. 10.1016/j.ecolmodel

[4] Gaughan A. E., Stevens F. R., Linard C., Jia P., & Tatem A. J. (2013). High Resolution Population Distribution Maps for Southeast Asia in 2010 and 2015. *PLoS ONE*, 8(2), e55882. 10.1371/journal.pone.0055882 23418469 PMC3572178

[5] Maantay J. A., Maroko A. R., & Herrmann C. (2007). Mapping Population Distribution in the Urban Environment: The Cadastral-based Expert Dasymetric System (CEDS). *Cartography and Geographic Information Science*, 34(2), 77–102. 10.1559/152304007781002190

[6] Kang C., Liu Y., Ma X., & Wu L. (2012). Towards Estimating Urban Population Distributions from Mobile Call Data. *Journal of Urban Technology*, 19(4), 3–21. 10.1080/10630732.2012.715479

[7] Liu Y. (2018). Introduction to land use and rural sustainability in China. *Land Use Policy*, 74, 1–4. 10.1016/j.landusepol.2018.01.032

[8] Yang J., Yang R., Chen M. H., Su C. H. J., Zhi Y., & Xi J. (2021). Effects of rural revitalization on rural tourism. *Journal of Hospitality and Tourism Management*, 47, 35–45.

[9] Liu X. H., Kyriakidis P. C., & Goodchild M. F. (2008). Population‐density estimation using regression and area‐to‐point residual kriging. *International Journal of Geographical Information Science*, 22(4), 431–447. 10.1080/13658810701492225

[10] Zhuo L., Ichinose T., Zheng J., Chen J., Shi P. J., & Li X. (2009). Modelling the population density of China at the pixel level based on DMSP/OLS non‐radiance‐calibrated night‐time light images. *International Journal of Remote Sensing*, 30(4), 1003–1018. 10.1080/01431160802430693

[11] Xu Z., & Ouyang A. (2017). The Factors Influencing China’s Population Distribution and Spatial Heterogeneity: a Prefectural-Level Analysis using Geographically Weighted Regression. *Applied Spatial Analysis and Policy*, 11(3), 465–480. 10.1007/s12061-017-9224-8

[12] Huang Y., Zhao C., Song X., Chen J., & Li Z. (2018). A semi-parametric geographically weighted (S-GWR) approach for modeling spatial distribution of population. *Ecological Indicators*, 85, 1022–1029.

[13] Yang J., Bao Y., Zhang Y., Li X., & Ge Q. (2018). Impact of accessibility on housing prices in Dalian city of China based on a geographically weighted regression model. *Chinese geographical science*, 28(3), 505–515.

[14] Graves P. E., & Mueser P. R. (2010). Examining the role of economic opportunity and amenities in explaining population redistribution. *Columbia Missouri University of Missouri Department of Economics Mar*, 37(2), 176–200.

[15] Zhang L., Lin X., Leng L., & Zeng Y. (2021). Spatial distribution of rural population from a climate perspective: Evidence from Jiangxi Province in China. *Plos one*, 16(3), e0248078. 10.1371/journal.pone.0248078 33662002 PMC7932106

[16] Geddes A. (1937). The Population of Bengal, Its Distribution and Changes: A Contribution to Geographical Method. *The Geographical Journal*, 89(4), 344. 10.2307/1785693

[17] Lei Y., Yang K., Wang B., Sheng Y., Bird B. W., Zhang G., et al. (2014). Response of inland lake dynamics over the Tibetan Plateau to climate change. *Climatic Change*, 125(2), 281–290. 10.1007/s10584-014-1175-3

[18] Miller H. J. (2004). Tobler’s First Law and Spatial Analysis. *Annals of the Association of American Geographers*, 94(2), 284–289. 10.1111/j.1467-8306.2004.09402005.x

[19] MORAN P. A. P. (1950). NOTES ON CONTINUOUS STOCHASTIC PHENOMENA. *Biometrika*, 37(1–2), 17–23. 10.1093/biomet/37.1–2.17 15420245

[20] Geary R. C. (1954). The Contiguity Ratio and Statistical Mapping. *The Incorporated Statistician*, 5(3), 115. 10.2307/2986645

[21] Stewart Fotheringham A., Charlton M., & Brunsdon C. (1996). The geography of parameter space: an investigation of spatial non-stationarity. *International Journal of Geographical Information Systems*, 10(5), 605–627. 10.1080/02693799608902100

[22] Brunsdon C., Fotheringham A. S., & Charlton M. (1999). Some Notes on Parametric Significance Tests for Geographically Weighted Regression. *Journal of Regional Science*, 39(3), 497–524. 10.1111/0022-4146.00146

[23] Tian Y., Yue T., Zhu L., & Clinton N. (2005). Modeling population density using land cover data. *Ecological Modelling*, 189(1–2), 72–88. 10.1016/j.ecolmodel.2005.03.012

[24] Ye X., Zhang Q., Liu J., Li X., & Xu C. (2013). Distinguishing the relative impacts of climate change and human activities on variation of streamflow in the Poyang Lake catchment, China. *Journal of Hydrology*, 494, 83–95. 10.1016/j.jhydrol.2013.04.036

[25] Zhang D. D., Lee H. F., Wang C., Li B., Zhang J., Pei Q., et al. (2011). Climate change and large-scale human population collapses in the pre-industrial era. *Global Ecology and Biogeography*, 20(4), 520–531. 10.1111/j.1466-8238.2010.00625.x

[26] Small C., & Cohen J. E. (2004). Continental Physiography, Climate, and the Global Distribution of Human Population. *Current Anthropology*, 45(2), 269–277. 10.1086/382255

[27] Appleby S. (2010). Multifractal Characterization of the Distribution Pattern of the Human Population. *Geographical Analysis*, 28(2), 147–160. 10.1111/j.1538-4632.1996.tb00926.x

[28] Bhaduri B., Bright E., Coleman P., & Urban M. L. (2007). LandScan USA: a high-resolution geospatial and temporal modeling approach for population distribution and dynamics. *GeoJournal*, 69(1–2), 103–117. 10.1007/s10708-007-9105-9

